# Urinary cadmium and endometriosis prevalence in a US nationally representative sample: results from NHANES 1999–2006

**DOI:** 10.1093/humrep/dead117

**Published:** 2023-07-24

**Authors:** Mandy S Hall, Nicole M Talge, Kristen Upson

**Affiliations:** Department of Epidemiology and Biostatistics, College of Human Medicine, Michigan State University, East Lansing, MI, USA; Department of Epidemiology and Biostatistics, College of Human Medicine, Michigan State University, East Lansing, MI, USA; Department of Epidemiology and Biostatistics, College of Human Medicine, Michigan State University, East Lansing, MI, USA

**Keywords:** endometriosis, cadmium, epidemiology, environmental health, cross-sectional study

## Abstract

**STUDY QUESTION:**

Is exposure to toxic metal cadmium associated with increased endometriosis prevalence among a nationally representative sample of the US population?

**SUMMARY ANSWER:**

Concentrations of urinary cadmium, a long-term biomarker (10–30 years) of cadmium exposure, were associated with an increased prevalence of endometriosis.

**WHAT IS KNOWN ALREADY:**

Cadmium exhibits estrogenic properties and may increase the risk of endometriosis, a gynecologic condition associated with substantial morbidity, for which estrogen has a central pathogenic role. Previous epidemiological studies of cadmium and endometriosis have yielded mixed results, with null, positive, and inverse associations being reported.

**STUDY DESIGN, SIZE, DURATION:**

We conducted a cross-sectional study using data from four cycles of the National Health and Nutrition Examination Survey (NHANES) 1999–2006.

**PARTICIPANTS/MATERIALS, SETTING, METHODS:**

The study population comprised participants aged 20–54 years who had an endometriosis diagnosis, available data on urinary cadmium, and a glomerular filtration rate ≥60 ml/min/1.73 m^2^ (unweighted n = 1647). Urinary cadmium was measured by inductively coupled plasma–mass spectrometry, and we used urinary creatinine concentrations and covariate-adjusted standardization to account for urinary dilution. Self-reported diagnosis of endometriosis was ascertained by interview. We examined the association between quartiles of urinary cadmium and endometriosis using log-binomial regression to estimate adjusted prevalence ratios (aPRs) and 95% CIs.

**MAIN RESULTS AND THE ROLE OF CHANCE:**

We observed twice the prevalence of endometriosis for participants with cadmium concentrations in the second quartile (versus the first quartile) (aPR 2.0, 95% CI: 1.1, 3.9) and the third quartile (versus the first quartile) (aPR 2.0, 95% CI: 1.1, 3.7). Our data also suggested a 60% increased prevalence of endometriosis with urinary cadmium concentrations in the fourth quartile (versus the first quartile) (aPR 1.6, 95% CI: 0.8, 3.2). In a sensitivity analysis, restricting the study population to premenopausal participants with an intact uterus and at least one ovary (unweighted n = 1298), stronger associations accompanied by wider CIs were observed.

**LIMITATIONS, REASONS FOR CAUTION:**

We were limited by the ascertainment of urinary cadmium and endometriosis diagnosis at a single time point, given the cross-sectional study design, and we relied on self-report of endometriosis diagnosis. However, urinary cadmium characterizes long-term exposure and findings from validation studies suggest that misclassification of self-reported endometriosis diagnosis may be minimal.

**WIDER IMPLICATIONS OF THE FINDINGS:**

This study suggests that cadmium is associated with an increased endometriosis prevalence. Given the substantial morbidity conferred by endometriosis and that the general population is ubiquitously exposed to cadmium, further research is warranted to confirm our findings.

**STUDY FUNDING/COMPETING INTEREST(S):**

This work was supported by the National Institute of Nursing Research (grant R00NR017191 to K.U.) of the National Institutes of Health. The authors report no conflict of interest.

**TRIAL REGISTRATION NUMBER:**

N/A.

## Introduction

Endometriosis is characterized by the presence of endometrial-like glands and stroma outside of the uterus. This condition, estimated to affect ∼10% of reproductive-age women (Giudice and Kao, 2004; Ghiasi *et al.*, 2020), is associated with substantial morbidity, including painful menstruation and chronic pelvic pain (Holt and Weiss, 2000). In addition, endometriosis is associated with adverse outcomes, such as infertility, ovarian cancer, cardiovascular disease, and cutaneous melanoma cancer (Fourquet *et al.*, 2010; Moradi *et al.*, 2014; Kvaskoff *et al.*, 2015). Despite negatively affecting health and well-being, the etiology of endometriosis remains unclear. However, estrogen is recognized to have a central role in its pathogenesis, as increased local production of estrogen promotes the survival, implantation, and proliferation of ectopic endometrium (Laganà *et al.*, 2019).

Environmental chemicals that exhibit estrogenic properties, such as the toxic metal cadmium, may increase the risk of endometriosis. Cadmium can bind to estrogen receptors and has been observed to increase endometrial proliferation in *in vitro* and *in vivo* studies (Garcia-Morales *et al.*, 1994; Stoica *et al.*, 2000; Johnson *et al.*, 2003; Silva *et al.*, 2013). The World Health Organization places cadmium in the top 10 chemicals of public health concern (WHO, 2020). Although there is no established level of cadmium detection that requires medical intervention in humans, cadmium has been observed to be associated with health problems at a low level of exposure, indicating that there is no ‘safe’ level of cadmium exposure (ATSDR, 2012; CDC, 2019). The general population is exposed to cadmium primarily through the inhalation of cigarette smoke and the consumption of contaminated food (Järup and Akesson, 2009). Cadmium is released into the environment through industrial processes, where it is absorbed by plants, including tobacco, leafy vegetables, root vegetables, grains, soybeans, and by aquatic organisms, such as shellfish (ATSDR, 2012). In humans, cadmium accumulates in the kidneys where it has a long biological half-life due to an inefficient excretion mechanism (Järup and Akesson, 2009).

Prior epidemiologic studies investigating cadmium and endometriosis have yielded mixed results. Most studies have reported a null association between blood cadmium (Heilier *et al.*, 2004; 2006; Pollack *et al.*, 2013; Silva *et al.*, 2013; Lai *et al.*, 2017) or urinary cadmium (Heilier *et al.*, 2006; Itoh *et al.*, 2008; Pollack *et al.*, 2013) and endometriosis, although two studies observed a positive (Jackson *et al.*, 2008) and inverse (Pollack *et al.*, 2013) association between blood cadmium and endometriosis. Both the approach to study population sampling and cadmium measurement likely contribute to the discrepant results across studies. As endometriosis can only be definitively diagnosed through surgical visualization (Brosens, 1997), most of the previous studies restricted the study population to patients undergoing gynecologic surgery (Itoh *et al.*, 2008; Pollack *et al.*, 2013; Silva *et al.*, 2013; Lai *et al.*, 2017). However, surgical patients without endometriosis may have a medical indication warranting surgery that is associated with cadmium exposure; thus, this comparison group of non-cases may have altered levels of cadmium, yielding biased estimates of the association. This bias may be avoided in studies employing a population-based sampling frame (Jackson *et al.*, 2008; Pollack *et al.*, 2013), with non-cases representing the frequency of cadmium exposure in the population that gave rise to the endometriosis cases. In addition, misclassification of exposure may occur from the biological sample used for cadmium measurement; cadmium measured in whole blood characterizes recent exposure over the prior 2–3 months, whereas cadmium measured in urine reflects long-term exposure over the past 10–30 years (Järup *et al.*, 1983; Järup and Akesson, 2009; ATSDR, 2012). Considering both the importance of study population sampling and cadmium measurement, the purpose of the present study was to investigate the association between urinary cadmium and endometriosis prevalence using a nationally representative sample of the US population.

## Materials and methods

### Data source and study design

We conducted a cross-sectional study using data from the National Health and Nutrition Examination Survey (NHANES) across four data cycles: 1999–2000, 2001–2002, 2003–2004, and 2005–2006 (n = 41 474). NHANES, a large population-based cross-sectional study, is conducted by the National Center for Health Statistics (NCHS) at the Center for Disease Control and Prevention (CDC) (Zipf *et al.*, 2013). Since the purpose of NHANES is to assess exposure to environmental chemicals as well as to monitor the health and nutritional status of the US population, a complex multistage sampling scheme is used to recruit a representative sample of the civilian, non-institutionalized US population. Participation in NHANES comprises two phases, a home interview and health examination, with data being collected by interview, physical examination, and laboratory testing (Zipf *et al.*, 2013). Questionnaires covering a range of topics were used during the household interview (e.g. demographics; household characteristics; medical conditions; physical activity) or during the mobile examination center (MEC) examination among a subsample of participants (e.g. reproductive health; tobacco smoking; alcohol consumption). Blood and urine samples were also collected at the MEC examination, along with urine pregnancy tests and body measurements as assessed by an NHANES examiner.

### Ethical approval

All participants included in the NHANES cycles completed the informed consent process (Zipf *et al.*, 2013). The NCHS Ethics Review Board at the CDC approved the data collection for NHANES. As the present analysis uses publicly de-identified datafiles, the Human Research Protection Program at Michigan State University deemed the present analyses to not involve human subjects.

### Urinary cadmium and creatinine measurement

Spot urine samples were collected at the MEC examination from one-third of the NHANES study population and measured for urinary cadmium concentrations (ng/ml) using inductively coupled plasma–mass spectrometry (ICP-MS) by the Division of Environmental Health Laboratory Science at the Centers for Disease Control and Prevention (CDC) (NCHS, 2008a,b). Urinary cadmium concentrations were corrected for interference from molybdenum oxide mathematically in cycles 1999–2002; for cycles 2003–2006, urinary cadmium concentrations were corrected at the testing laboratory. For NHANES 1999–2004, the limit of detection (LOD) was 0.06 ng/ml, whereas the LOD was 0.042 ng/ml in NHANES 2005–2006 (NCHS, 2003, 2008a,b). Since the percent of observations below the LOD were 5% and 8% for these respective cycles, urinary cadmium concentrations below the LOD were replaced with the LOD divided by the square root of two (Lubin *et al.*, 2004).

To correct urinary cadmium concentrations for urinary dilution, we used data on urinary creatinine concentrations (mg/dl) measured using a Jaffe rate reaction method with the Beckman Synchron CX3 Clinical analyzer (Beckman Instruments, Inc., Brea, CA, USA) at the University of Minnesota (NCHS, 2008a,b). The lower LOD was 1 mg/dl; values <10 mg/dl and >400 mg/dl were reanalyzed (NCHS, 2008a,b). None of the participants had urinary creatinine concentrations below the LOD. Using the urinary creatinine concentrations, we accounted for urinary dilution by employing covariate-adjusted standardization of the urinary cadmium concentrations (O’Brien *et al.*, 2016). Briefly, this approach entails standardizing the urinary cadmium concentrations by the estimated proportion of urinary creatinine reflecting hydration. Among the participants included in the present analyses (described below), we fitted a model of the natural logarithm of urinary creatinine as a function of covariates that affect urinary creatinine outside of hydration (Boeniger *et al.*, 1993; Barr *et al.*, 2005). The covariates that were selected were age at sample collection (years, continuous), BMI (weight (kg)/height (m)^2^, continuous), waist circumference (cm, continuous), smoking status (never smoker, former smoker, current smoker of <20 cigarettes/day, current smoker of ≥20 cigarettes/day), alcohol consumption (never, former, current), reported history of diabetes when not pregnant (yes/borderline, no), history of liver disease (yes, no), history of hypertension (yes, no), history of thyroid disease (yes/no), and positive pregnancy test result at sample collection (yes, no). For the covariates in which the participant reported ‘don’t know’ (alcohol (unweighted n = 1), history of hypertension (unweighted n = 3), and history of liver disease (unweighted n = 1)), the variables were coded as ‘no’. Using the fitted model, we estimated the linear prediction of the log urinary creatinine values and exponentiated these predicted log values to obtain the fitted urinary creatinine values. We then calculated the ratio of observed urinary creatinine to fitted urinary creatinine and divided the urinary cadmium concentrations by this ratio.

As the dose–response relationship between an endocrine-disrupting chemical and disease may not follow a monotonic linear relationship, we categorized the covariate-adjusted standardized urinary cadmium concentrations (ng/ml) in quartiles to allow for flexible modeling of the dose–response relationship with endometriosis prevalence (Birnbaum and Jung, 2011). The quartiles were determined using the distribution of covariate-adjusted standardized urinary cadmium concentrations in the study population.

### Endometriosis

In four NHANES cycles (1999–2000, 2001–2002, 2003–2004, and 2005–2006), participants coded as female by the NHANES field household interviewer were asked questions during the MEC examination on reproductive health. History of endometriosis diagnosis was only collected in NHANES among females ages 20–54 years. Participants were asked, ‘Has a doctor or other health professional ever told you that you had endometriosis? Endometriosis is a disease in which the tissue that forms the lining of the uterus/womb attaches to other places, such as the ovaries, fallopian tubes etc.’. If a participant responded yes, they were asked age at diagnosis. Using this information, we created a binary variable on history of endometriosis diagnosis (no, yes). Given that information on gender collected during the household interview was limited to male or female and was only asked by the field interviewer ‘if not obvious’, we used gender-inclusive language when describing the NHANES participants (NCHS, 1999; Rioux *et al.*, 2022).

### Participants

We limited our study population to participants aged 20–54 years with available information on endometriosis diagnosis and urinary cadmium concentrations (unweighted n = 1750). We included participants regardless of menopausal, hysterectomy, and bilateral oophorectomy status at time of NHANES interview to minimize selection bias. In the cross-sectional setting, selection bias could occur from excluding participants on reproductive events (menopause, hysterectomy, and bilateral oophorectomy) that happened after the development of endometriosis and that may be associated with cadmium exposure (Pollack *et al.*, 2014; Lee *et al.*, 2018; Upson *et al.*, 2021). Reproductive events could occur after endometriosis development since (i) urinary cadmium is a long-term biomarker that characterizes exposure over the last 10–30 years (Järup and Akesson, 2009) and (ii) there was a median of 10 years between endometriosis diagnosis and NHANES interview in this study sample. We also excluded participants with an estimated glomerular filtration rate <60 ml/min/1.73 m^2^, indicating chronic kidney disease (unweighted n = 103), as reduced renal function could affect the elimination of urinary cadmium. Glomerular filtration rate was estimated using the Chronic Kidney Disease Epidemiology Collaboration (CKD-EPI) creatinine equation refit without the race variable (Delgado *et al.*, 2022), using serum creatinine concentrations (mg/dl) measured by the modified Jaffe rate method for cycles 1999–2002 and the original Jaffe rate method for cycles 2003–2006 (NCHS, 2010). After exclusions, a total of 1647 participants (unweighted) were included in the present analyses.

### Statistical analyses

We conducted descriptive statistics to compare sociodemographic and reproductive health characteristics between those with and without endometriosis. We then examined the association between covariate-adjusted standardized urinary cadmium quartiles and endometriosis using log-binomial regression to estimate prevalence ratios (PRs) and 95% CIs. We selected covariates for adjustment a priori using a directed acyclic graph (Supplementary Fig. S1). All analyses were adjusted for age at interview (continuous), education (≤high school education, some college or associate degree, college graduate or above), and smoking status (never smoker, former smoker, current smoker of <20 cigarettes/day, current smoker of ≥20 cigarettes/day). We also adjusted for urinary creatinine (continuous) to optimally account for urinary dilution when using covariate-adjusted standardization (O’Brien *et al.*, 2016). Since <1% of data on covariates were missing, we used available data in the analyses. We did not adjust for other metal biomarkers available in NHANES (urinary: barium, beryllium, cobalt, cesium, lead, platinum, antimony, thallium, tungsten; blood: lead, mercury, and iron) as these biomarkers characterize shorter-term exposure (Flora, 2014) and some biomarkers, such as blood lead, are affected by bone changes after menopause and would not characterize metal body burden at the time of endometriosis development (Silbergeld *et al.*, 1988; Symanski and Hertz-Picciotto, 1995; Hernandez-Avila *et al.*, 2000; Nash, 2004). To explore the functional form of the dose–response relationship between urinary cadmium and endometriosis prevalence, we repeated our analyses using restricted cubic splines, with knots at the 5th, 35th, 65th, and 95th percentiles of the weighted urinary cadmium distribution. We designated the first percentile as the reference urinary cadmium concentration.

We conducted several sensitivity analyses. First, to evaluate the impact of including participants in the study population regardless of menopausal, hysterectomy, and bilateral oophorectomy status at time of NHANES interview on our results, we repeated the analyses after restricting the study population to premenopausal participants with an intact uterus and at least one ovary (75% of the original study population; unweighted n = 1298). Second, to understand the impact of the urinary dilution method on the results, we repeated the analyses accounting for urinary dilution using the standardization method; we divided the cadmium concentrations (ng/ml) by urinary creatinine concentrations (mg/dl) and multiplied by 100 to obtain standardized cadmium concentrations (μg/g). Third, since we did not adjust for parity in the main analyses as it may be affected by endometriosis, we repeated the analyses additionally adjusting for parity. Lastly, given the potential for residual confounding by cigarette smoking since the inhalation of cigarette smoke is a strong contributor to cadmium body burden (ATSDR, 2012), we repeated the analyses restricting the study population to never smokers.

Given the median of 10 years between endometriosis diagnosis and NHANES interview in the study sample, we conducted a descriptive analysis to examine whether the urinary cadmium concentrations varied by years since endometriosis diagnosis. We estimated the geometric mean (GM) concentrations of standardized urinary cadmium concentrations, adjusted for age, education, and smoking status, at ≤5, >5–10, >10–15, and >15 years after endometriosis diagnosis. We conducted this descriptive analysis since only a few endometriosis cases (unweighted n = 35) were diagnosed in the 5 years before interview, limiting our ability to conduct a sensitivity analysis considering only recently diagnosed cases. Analyses were performed with SAS version 9.4 (Cary, NC, USA) and STATA version 15.1 (Stata Corp LLC, College Station, TX, USA) using survey procedure commands to account for the complex survey sampling design and weighting of data.

## Results

In this study population, the weighted prevalence of endometriosis was 9.4% (unweighted n = 108). Those with a history of endometriosis diagnosis tended to be older, non-Hispanic White, have greater educational attainment (college graduate or above), and have a higher poverty income ratio (>3.5, indicating higher income status) than those without a history of endometriosis diagnosis (Table 1). Compared to non-cases, endometriosis cases also tended to have a history of smoking and be current consumers of alcohol. Cases were also more frequently nulliparous, menopausal, and reported a history of hysterectomy and/or bilateral oophorectomy compared to those without endometriosis. The number of years between endometriosis diagnosis and NHANES interview ranged from 0 to 31 years, with the median number of years being 10 years (interquartile range: 4–15).

**Table 1.** Participant characteristics by diagnosis of endometriosis, National Health and Nutrition Examination Survey (NHANES), 1999–2006.

	Endometriosis	
Participant characteristic	Yes	No
	(n = 108)^a^	(n = 1539)^a^
	%^b^	%^b^
Age at sample collection, years		
20–29	7	27
30–39	37	28
40–49	48	31
50–54	8	14
Race/Ethnicity		
Mexican American	2	9
Other Hispanic	3	6
Non-Hispanic White	90	68
Non-Hispanic Black	6	12
Other/more than one race	1	4
Education		
≤HS graduate	10	17
HS graduate	31	23
Some college or associate degree	27	34
College graduate or above	31	26
Poverty income ratio		
≤1.3	17	23
>1.3–3.5	29	36
>3.5	53	41
Smoking status		
Never smoker	53	58
Former smoker	18	19
Current smoker, cigarettes/day		
<20	17	15
≥20	12	9
Alcohol consumption		
Never	5	15
Former	21	18
Current	74	67
BMI (kg/m)^c^		
<25	44	39
25–<30	26	27
30–<35	12	16
≥35	19	17
Menarche age (years)		
≤10	10	9
11	10	14
12	34	26
13	24	25
≥14	23	27
Number of live births		
0	31	26
1	16	20
2	27	27
≥3	27	27
Menopausal status		
No	58	80
Yes	42	20
Hysterectomy		
No	55	90
Yes	45	10
Bilateral oophorectomy		
No	76	96
Yes	24	4

HS, high school.

a Unweighted sample size.

b Weighted percentage using NHANES sampling design.

c Using height and weight measured during the NHANES mobile exam component.

The GM and distribution of urinary cadmium concentrations were slightly higher for participants with a history of endometriosis diagnosis (0.30 μg/g, 95% CI: 0.27–0.34) than participants without such a history (0.25 μg/g, 95% CI: 0.24–0.26). After multivariable adjustment, we observed twice the prevalence of endometriosis for participants with cadmium concentrations in the second quartile (PR 2.0, 95% CI: 1.1, 3.9) and the third quartile (PR 2.0, 95% CI: 1.1, 3.7) compared to the first quartile (Table 2). Our data suggested a 60% increased prevalence of endometriosis with urinary cadmium concentrations in the fourth quartile versus the first quartile (PR 1.6, 95% CI: 0.8, 3.2). We observed a similar dose–response relationship when using restricted cubic splines to characterize urinary cadmium concentrations (Supplementary Fig. S2).

**Table 2.** Adjusted prevalence ratios (aPRs) and 95% CI for the association between quartiles of urinary cadmium and endometriosis among participants aged 20–54 years (unweighted n = 1647), National Health and Nutrition Examination Survey, 1999–2006.

	Endometriosis		
	Yes	No	
	(n = 108)^a^	(n = 1539)^a^	
	%^b^	%^b^	PR (95% CI)^c^
Urinary cadmium (ng/ml)^d^^,^^e^			
Quartile 1: <0.15	13	26	1.0 Reference
Quartile 2: 0.15–<0.23	29	25	2.0 (1.1, 3.9)
Quartile 3: 0.23–<0.38	30	25	2.0 (1.1, 3.7)
Quartile 4: ≥0.38	28	25	1.6 (0.8, 3.2)

PR, prevalence ratio.

a Unweighted n.

b Weighted percent.

c Adjusted for age at screening (continuous), smoking (never, former, current smoker of <20 cigarettes/day, current smoker of ≥20 cigarettes/day), education (≤high school education, some college or associate degree, college graduate or above), and urinary creatinine (continuous).

d Covariate-adjusted standardization of urinary cadmium concentrations by dividing urinary cadmium concentrations by the ratio of the observed and predicted urinary creatinine concentrations. Predicted urinary creatinine concentrations were estimated by fitting a model for natural logarithm-transformed urinary creatinine as a function of age at sample collection, BMI, waist circumference, smoking status, alcohol consumption, history of diabetes, liver disease, hypertension, or thyroid disease, and positive pregnancy test result.

e For NHANES participants with and without a history of endometriosis diagnosis, covariate-adjusted standardized concentrations of urinary cadmium were missing for n = 1 (unweighted) and n = 32 (unweighted) participants, respectively.

When we restricted the study population to those who did not have a history of menopause, hysterectomy, or bilateral oophorectomy, we observed prevalence ration (PR) estimates that were stronger in magnitude but with wider CIs compared to those in the main analyses (Table 3). For example, those with urinary cadmium concentrations in the third quartile had three times of the prevalence of endometriosis (PR 3.0, 95% CI: 1.3, 6.9) than those with urinary cadmium concentrations in the first quartile.

**Table 3.** Adjusted prevalence ratios (aPRs) and 95% CI for the association between quartiles of urinary cadmium and endometriosis among premenopausal participants ages 20–54 years with intact uterus and at least one ovary (unweighted n = 1298), National Health and Nutrition Examination Survey, 1999–2006.

	Endometriosis		
	Yes	No	
	(n = 61)^a^	(n = 1237)^a^	
	%^b^	%^b^	PR (95% CI)^c^
Urinary cadmium (ng/ml)^d^^,^^e^			
Quartile 1: <0.15	12	29	1.0 Reference
Quartile 2: 0.15–<0.23	28	26	2.4 (1.1, 5.5)
Quartile 3: 0.23–<0.38	35	24	3.0 (1.3, 6.9)
Quartile 4: ≥0.38	25	21	2.6 (0.9, 7.1)

PR, prevalence ratio.

a Unweighted n.

b Weighted percent.

c Adjusted for age at screening (continuous), smoking (never, former, current smoker of <20 cigarettes/day, current smoker of ≥20 cigarettes/day), education (≤high school education, some college or associate degree, college graduate or above), and urinary creatinine (continuous).

d Covariate-adjusted standardization of urinary cadmium concentrations by dividing urinary cadmium concentrations by the ratio of the observed and predicted urinary creatinine concentrations. Predicted urinary creatinine concentrations were estimated by fitting a model for natural logarithm-transformed urinary creatinine as a function of age at sample collection, BMI, waist circumference, smoking status, alcohol consumption, history of diabetes, liver disease, hypertension, or thyroid disease, and positive pregnancy test result.

e For NHANES participants with and without a history of endometriosis diagnosis, covariate-adjusted standardized concentrations of urinary cadmium were missing for n = 1 (unweighted) and n = 23 (unweighted) participants, respectively.

In our sensitivity analysis using the standardization method to correct for urinary dilution (Supplementary Table S1) or additionally adjusting for parity (Supplementary Table S2), we observed results similar to that of the main analyses. We observed results that were attenuated in magnitude compared to the main analysis when we restricted the study population to never smokers (Supplementary Table S3). Among the endometriosis cases, the adjusted GM urinary cadmium concentrations were similar across years since diagnosis, although the GM cadmium concentration was slightly higher at >10–15 years after diagnosis (0.36 μg/g) and slightly lower at >15 years after diagnosis (0.28 μg/g) (Supplementary Table S4).

## Discussion

Using data from a nationally representative sample of the US population, we observed that urinary cadmium was associated with increased prevalence of endometriosis. The observed association is biologically plausible. The leading theories of endometriosis pathogenesis postulate that ectopic endometrial-like glands and stroma originate from metaplasia of local tissue or from transplanted eutopic endometrium (Laganà *et al.*, 2019). Increased local production of estrogen promotes the survival, implantation, and proliferation of ectopic endometriotic lesions (Laganà *et al.*, 2019). As estrogen is integral to endometriosis pathogenesis and endometriotic lesion progression, exposure to cadmium, a metalloestrogen, may increase the risk of endometriosis. The estrogenic properties of cadmium have been demonstrated in *in vitro* studies of human breast cancer cells, by binding to estrogen receptors and increasing cellular proliferation, with its effects being inhibited by antiestrogens (Garcia-Morales *et al.*, 1994; Stoica *et al.*, 2000). Cadmium has also exhibited effects on uterine tissue. Using the Sprague-Dawley rat model, female ovariectomized rats treated with cadmium had a 1.9-fold increase in uterine wet weight with endometrial proliferation (Johnson *et al.*, 2003). An *in vitro* study of endometrial stromal cells from the eutopic endometrium treated with cadmium observed increased proliferation of endometrial stromal cells among women with endometriosis compared to women without the condition (Silva *et al.*, 2013). In addition to operating through hormonal pathways, cadmium may also operate through inflammatory and immunologic processes given the multifactorial nature of endometriosis (Pollack *et al.*, 2014; Laganà *et al.*, 2019).

Endocrine-disrupting chemicals, like cadmium, may produce different biological effects based on the level of exposure (Birnbaum and Jung, 2011). For example, the biological response may increase as the exposure to endocrine-disrupting chemicals increases. However, there may be a threshold at which exposure to endocrine-disrupting chemicals can saturate hormone receptors, downregulate the receptors, and cause decreased sensitivity of the cell to the endocrine-disrupting chemical (Zoeller *et al.*, 2012). Similarly, we observed a non-monotonic dose–response relationship between cadmium and endometriosis prevalence with a positive association observed with the second and third quartiles of cadmium concentrations, compared to the first quartile.

Our observation of a positive association between urinary cadmium and endometriosis contrasts with five prior studies that were conducted among surgical patients (Heilier *et al.*, 2006; Itoh *et al.*, 2008; Pollack *et al.*, 2013; Silva *et al.*, 2013; Lai *et al.*, 2017). Those studies reported null associations with blood and urinary cadmium and endometriosis (Heilier *et al.*, 2006; Itoh *et al.*, 2008; Pollack *et al.*, 2013; Silva *et al.*, 2013; Lai *et al.*, 2017), with the exception of one study that reported an inverse association between blood cadmium and endometriosis (Pollack *et al.*, 2013). Given that a medical indication is warranted for surgical investigation, it is possible that surgical patients without endometriosis had altered cadmium levels, obscuring the observed association. Only two prior studies investigated the association using a population-based sampling frame. Jackson *et al* (2008) used NHANES 1999–2001 data and reported a dose–response association between blood cadmium, indicative of recent exposure in the prior 2–3 months, and endometriosis (second tertile versus first: adjusted odds ratio (OR) 1.94, 95% CI: 0.73, 5.18; third tertile versus first: adjusted OR 3.39, 95% CI: 1.37, 8.40) (Jackson *et al.*, 2008). The other study screened 114 women by magnetic resonance imaging (MRI) for endometriosis and detected primarily ovarian endometriosis in 14 individuals (Pollack *et al.*, 2013). That study, most likely due to being underpowered, did not detect an association between cadmium measured in blood or urine and endometriosis. Hence, the discrepant results across studies are likely due to differences in the sampling frame, sample size, and characterization of cadmium exposure.

Our study had several limitations. First, given the cross-sectional study design, urinary cadmium concentrations and history of endometriosis diagnosis were both ascertained at the time of NHANES participation. However, urinary cadmium characterizes long-term exposure over the past 10–30 years due to the long elimination half-life from the kidneys, where it is deposited after exposure (Järup and Akesson, 2009). The use of a long-term biomarker of cadmium lends support to our assumption that exposure preceded the outcome, despite participants with endometriosis being diagnosed a median 10 years before NHANES interview.

Second, it is possible that the observed association between urinary cadmium and endometriosis prevalence may be confounded by another co-occurring gynecological condition, uterine fibroids. In NHANES, history of uterine fibroid diagnosis was collected by self-report, limiting our ability to account for this condition in the analyses. The use of self-report substantially misclassifies those without fibroids as compared to ultrasound screening as only a subset of fibroids are clinically diagnosed and uterine fibroids are exceedingly common; an ultrasound-based study reported a high cumulative incidence of fibroids nearly reaching 70% among White women and exceeding 80% among Black women by age 50 (Baird *et al.*, 2003). Additionally, endometriosis may co-occur with other gynecological conditions such as polycystic ovary syndrome, infertility, adenomyosis, and pregnancy loss, which may also be associated with exposure to cadmium (Pollack *et al.*, 2014). However, information on these gynecological conditions were not collected in NHANES 1999–2006. Third, the possibility exists that residual confounding by tobacco smoking, a substantial contributor to cadmium exposure, may explain the positive association observed in our study. In our sensitivity analysis conducted among never smokers, the magnitude of the association between urinary cadmium and endometriosis was attenuated and accompanied with wide CIs. However, these results likely reflect statistical instability due to the small sample size and only 61 endometriosis cases. The persistence of the positive association in the sensitivity analysis suggests that residual confounding by tobacco smoking did not explain the observed association between urinary cadmium concentrations and endometriosis in the main analyses.

Fourth, we relied on self-report of endometriosis diagnosis. Although endometriosis can only be definitively diagnosed by surgical visualization (Brosens, 1997) and individuals are likely to remember a surgical procedure, information was not collected in NHANES as to whether the diagnosis occurred by surgery or by other means, such as examination or imaging, which may contribute to the misclassification of cases. However, in a validation study across four large epidemiological cohorts, including the Black Women’s Health Study (BWHS) (n = 59 001), French Teachers’ Cohort (E3N) (n = 98 995), Growing Up Today Study (GUTS) (n = 15 044), and Nurses’ Health Study II (NHSII) (n = 116 429), 85% (range: 75–97%) of self-reported endometriosis cases diagnosed by surgery were confirmed by medical record review (Shafrir *et al.*, 2021). The presence of undiagnosed endometriosis among non-cases is also possible. That said, the prevalence of primarily ovarian endometriosis (11%) detected by MRI among women from the general population in the Endometriosis, Natural History, Diagnosis, and Outcomes Study (Buck Louis *et al.*, 2011; Laganà *et al.*, 2019) suggests that the misclassification of non-cases may not have been substantial in our study.

Despite these limitations, the key strength of our study was the sampling frame of the NHANES, a representative sample of the civilian, non-institutionalized US population. This mitigates the impact of selection bias that can occur when the study population is restricted to those undergoing surgery. Surgical patients have a medical indication warranting surgery, and cadmium exposure, given its estrogenic properties, may plausibly be associated with other gynecological conditions. Thus, in studies comprised of surgical patients, the comparison group of non-cases may have altered levels of cadmium. In contrast, the use of a population-based sampling frame, such as that in the present study, allows non-cases to represent the frequency of cadmium exposure among the underlying population that gives rise to the cases.

Another strength of our study was the inclusion of participants who were postmenopausal or had surgical removal of the uterus or ovaries. We selected the study population in this manner, given the cross-sectional study design, to minimize selection bias that could occur from excluding individuals on events that may be related to endometriosis diagnosis or management or due to cadmium exposure. In our sensitivity analysis restricted to premenopausal individuals with an intact uterus and at least one ovary, we observed associations between urinary cadmium and endometriosis that were larger in magnitude compared to the main analyses. Lastly, given the measurement of cadmium in urine, we carefully accounted for urinary dilution using covariate-adjusted standardization. This method was developed to avoid sources of bias that may arise from commonly used approaches to address urinary dilution, including standardization (O’Brien *et al.*, 2016).

In conclusion, the results from our study suggest that long-term exposure to cadmium is associated with an increased prevalence of endometriosis. Given the substantial morbidity conferred by endometriosis and that the general population is ubiquitously exposed to cadmium, further research is warranted to confirm our findings.

## Supplementary Material

dead117_Supplementary_Figure_S1

dead117_Supplementary_Figure_S2

dead117_Supplementary_Table_S1

dead117_Supplementary_Table_S2

dead117_Supplementary_Table_S3

dead117_Supplementary_Table_S4

## Data Availability

The data were derived from sources in the public domain: NHANES https://wwwn.cdc.gov/nchs/nhanes/default.aspx.
