# Supplementary material for: Urinary cadmium and endometriosis prevalence in a US nationally representative sample: results from NHANES 1999–2006
Source: Hum Reprod. 2023 Jul 24;38(9):1835–42. doi: 10.1093/humrep/dead117 (PMC10477936; doi:10.1093/humrep/dead117)
Supplement: dead117_Supplementary_Figure_S1 [file dead117_supplementary_figure_s1.pdf]

OXFORD
human reproduction

*Human Reproduction*, 2023, 1–1
https://doi.org/10.1093/humrep/dead117
Supplementary Data

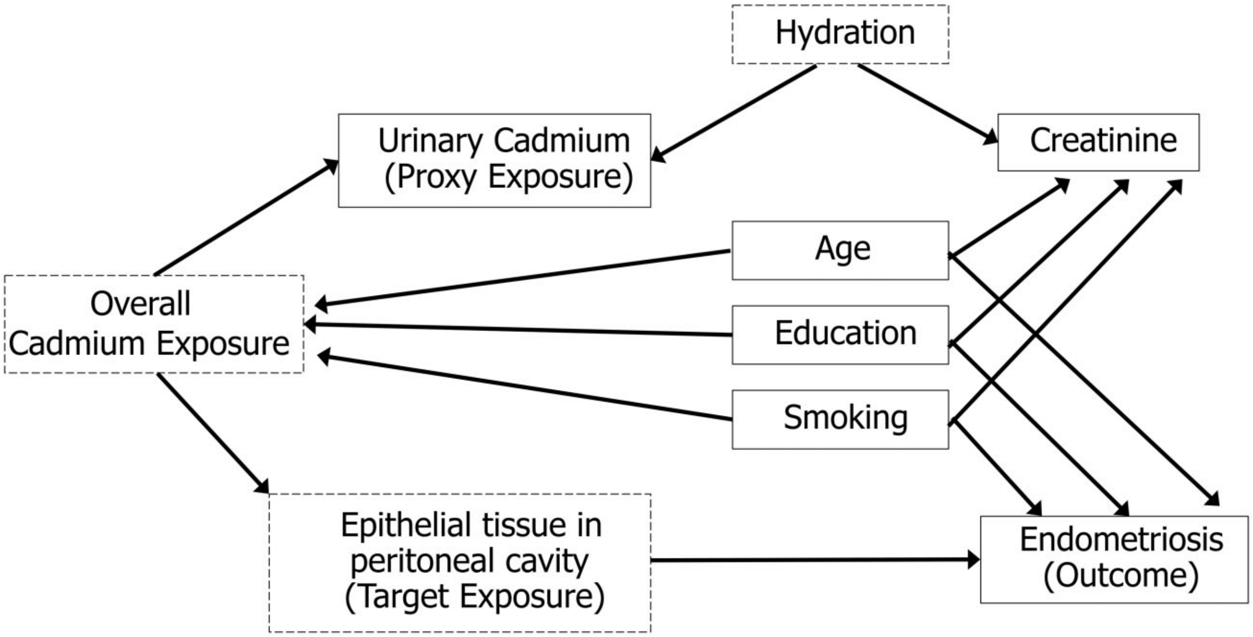


**Supplementary Figure S1. Directed acyclic graph (DAG) of the hypothesized casual pathway between urinary cadmium and endometriosis, National Health and Nutrition Examination Survey, 1999–2006.** Solid lines indicate observed variables; dashed lines indicate unobserved variables.
