# Supplementary material for: Urinary cadmium and endometriosis prevalence in a US nationally representative sample: results from NHANES 1999–2006
Source: Hum Reprod. 2023 Jul 24;38(9):1835–42. doi: 10.1093/humrep/dead117 (PMC10477936; doi:10.1093/humrep/dead117)
Supplement: dead117_Supplementary_Figure_S2 [file dead117_supplementary_figure_s2.pdf]

Human Reproduction, 2023, 1–1
https://doi.org/10.1093/humrep/dead117
Supplementary Data

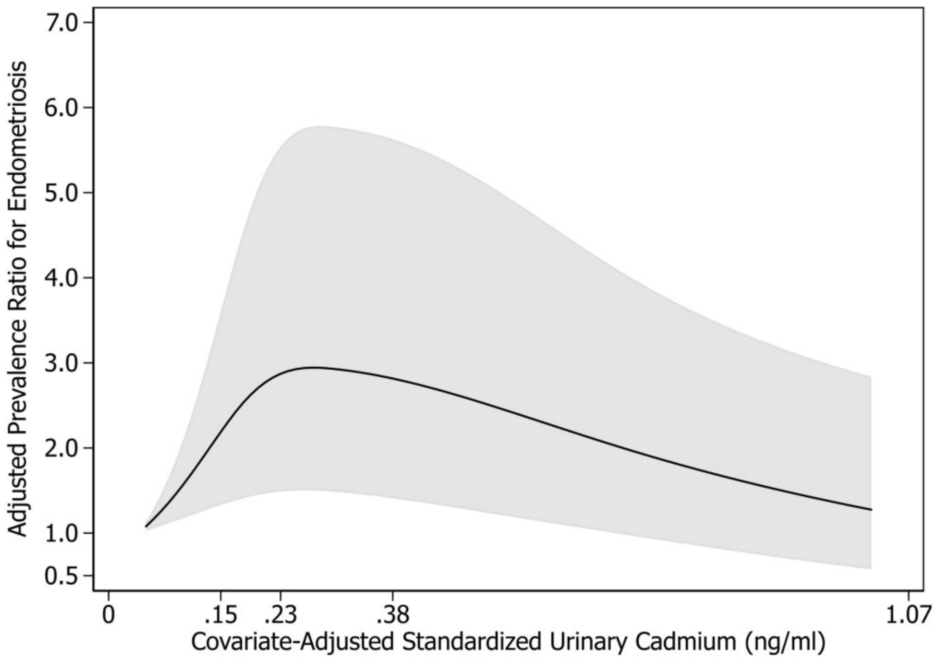

Supplementary Figure S2. Adjusted prevalence ratios and 95% CI for the association between quartiles of urinary cadmium and endometriosis using restricted cubic spline, National Health and Nutrition Examination Survey, 1999–2006. The solid line indicates the adjusted prevalence ratio; the shaded area represents the 95% CI.
