# Supplementary material for: Urinary cadmium and endometriosis prevalence in a US nationally representative sample: results from NHANES 1999–2006
Source: Hum Reprod. 2023 Jul 24;38(9):1835–42. doi: 10.1093/humrep/dead117 (PMC10477936; doi:10.1093/humrep/dead117)
Supplement: dead117_Supplementary_Table_S1 [file dead117_supplementary_table_s1.pdf]

OXFORD
human reproduction
Human Reproduction, 2023, 1–1
https://doi.org/10.1093/humrep/dead117
Supplementary Data

Supplementary Table S1. Adjusted prevalence ratios (aPRs) and 95% CI for the association between quartiles of urinary cadmium and endometriosis among participants aged 20–54 years (unweighted n = 1647), using standardized cadmium concentration, National Health and Nutrition Examination Survey, 1999–2006.

	Endometriosis		
	**Yes (n = 108)[a] %[b]**	**No (n = 1539)[a] %[b]**	**PR (95% CI)[c]**
Standardized urinary cadmium (μg/g)[d]			
Quartile 1: <0.16	12	26	1.0 Reference
Quartile 2: 0.16–<0.26	35	24	2.7 (1.3, 5.3)
Quartile 3: 0.26–<0.42	23	25	1.5 (0.7, 3.2)
Quartile 4: ≥0.42	30	24	1.8 (0.8, 4.0)

PR, prevalence ratio.
[a] Unweighted n.
[b] Weighted percent.
[c] Adjusted for age at screening (continuous), smoking (never, former, current smoker of <20 cigarettes/day, current smoker of ≥20 cigarettes/day), and education (≤high school education, some college or associate degree, college graduate or above).
[d] Urinary cadmium concentrations (ng/ml) were divided by urinary creatinine concentrations (mg/dl) and multiplied by 100 to obtain standardized cadmium concentrations (μg/g).
