# Supplementary material for: Urinary cadmium and endometriosis prevalence in a US nationally representative sample: results from NHANES 1999–2006
Source: Hum Reprod. 2023 Jul 24;38(9):1835–42. doi: 10.1093/humrep/dead117 (PMC10477936; doi:10.1093/humrep/dead117)
Supplement: dead117_Supplementary_Table_S2 [file dead117_supplementary_table_s2.pdf]

OXFORD

human reproduction

Human Reproduction, 2023, 1–1

https://doi.org/10.1093/humrep/dead117

Supplementary Data

Supplementary Table S2. Adjusted prevalence ratios (aPRs) and 95% CI for the association between quartiles of urinary cadmium and endometriosis among participants aged 20–54 years (unweighted n = 1647), additionally adjusting for parity, National Health and Nutrition Examination Survey, 1999–2006.

	Endometriosis		
	**Yes (n = 108)[a] %[b]**	**No (n = 1539)[a] %[b]**	**PR (95% CI)[c]**
Urinary cadmium (ng/ml)[d,e]			
Quartile 1: <0.15	13	26	1.0 Reference
Quartile 2: 0.15–<0.23	29	25	2.0 (1.0, 3.9)
Quartile 3: 0.23–<0.38	30	25	1.9 (1.0, 3.5)
Quartile 4: ≥0.38	28	25	1.5 (0.8, 3.1)

PR, prevalence ratio.

[a] Unweighted n.

[b] Weighted percent.

[c] Adjusted for age at screening (continuous), smoking (never, former, current smoker of <20 cigarettes/day, current smoker of ≥20 cigarettes/day), education (≤high school education, some college or associate degree, college graduate or above), urinary creatinine (continuous), and parity (0, 1, 2, ≥3 births).

[d] Covariate-adjusted standardization of urinary cadmium concentrations by dividing urinary cadmium concentrations by the ratio of the observed and predicted urinary creatinine concentrations. Predicted urinary creatinine concentrations were estimated by fitting a model for natural logarithm-transformed urinary creatinine as a function of age at sample collection, BMI, waist circumference, smoking status, alcohol consumption, history of diabetes, liver disease, hypertension, or thyroid disease, and positive pregnancy test result.

[e] For NHANES participants with and without a history of endometriosis diagnosis, covariate-adjusted standardized concentrations of urinary cadmium were missing for n = 1 (unweighted) and n = 32 (unweighted) participants, respectively.
