# Supplementary material for: Urinary cadmium and endometriosis prevalence in a US nationally representative sample: results from NHANES 1999–2006
Source: Hum Reprod. 2023 Jul 24;38(9):1835–42. doi: 10.1093/humrep/dead117 (PMC10477936; doi:10.1093/humrep/dead117)
Supplement: dead117_Supplementary_Table_S3 [file dead117_supplementary_table_s3.pdf]

OXFORD
human reproduction
Human Reproduction, 2023, 1–1
https://doi.org/10.1093/humrep/dead117
Supplementary Data

Supplementary Table S3. Adjusted prevalence ratios (aPRs) and 95% CI for the association between quartiles of urinary cadmium and endometriosis among participants aged 20–54 years with no smoking history (unweighted n = 1013), National Health and Nutrition Examination Survey, 1999–2006.

	Endometriosis		
	Yes (n = 61)[a] %[b]	No (n = 952)[a] %[b]	PR (95% CI)[c]
Urinary cadmium (ng/ml)[d,e]			
Quartile 1: <0.15	23	31	1.0 Reference
Quartile 2: 0.15–<0.23	34	28	1.6 (0.8, 3.2)
Quartile 3: 0.23–<0.38	24	26	1.1 (0.5, 2.7)
Quartile 4: ≥0.38	20	14	1.6 (0.7, 3.9)

PR, prevalence ratio.
[a] Unweighted n.
[b] Weighted percent.
[c] Adjusted for age at screening (continuous), education (≤high school education, some college or associate degree, college graduate or above), and urinary creatinine (continuous).
[d] Covariate-adjusted standardization of urinary cadmium concentrations by dividing urinary cadmium concentrations by the ratio of the observed and predicted urinary creatinine concentrations. Predicted urinary creatinine concentrations were estimated by fitting a model for natural logarithm-transformed urinary creatinine as a function of age at sample collection, BMI, waist circumference, smoking status, alcohol consumption, history of diabetes, liver disease, hypertension, or thyroid disease, and positive pregnancy test result.
[e] For NHANES participants with and without a history of endometriosis diagnosis, covariate-adjusted standardized concentrations of urinary cadmium were missing for n = 0 (unweighted) and n = 16 (unweighted) participants, respectively.
