# Supplementary material for: Urinary cadmium and endometriosis prevalence in a US nationally representative sample: results from NHANES 1999–2006
Source: Hum Reprod. 2023 Jul 24;38(9):1835–42. doi: 10.1093/humrep/dead117 (PMC10477936; doi:10.1093/humrep/dead117)
Supplement: dead117_Supplementary_Table_S4 [file dead117_supplementary_table_s4.pdf]

OXFORD
human reproduction
Human Reproduction, 2023, 1–1
https://doi.org/10.1093/humrep/dead117
Supplementary Data

Supplementary Table S4. Adjusted geometric mean standardized urinary cadmium concentrations and accompanying 95% CIs by years since endometriosis diagnosis among participants with a self-reported diagnosis of endometriosis ages 20–54 years (unweighted n = 108), National Health and Nutrition Examination Survey, 1999–2006.

	Endometriosis cases (n = 108)[a]	Urinary cadmium (μg/g)[b]
	%[c]	GM (95% CI)[d]
Years since endometriosis diagnosis		
≤5	32	0.30 (0.25–0.36)
>5–10	20	0.32 (0.25–0.41)
>10–15	25	0.36 (0.28–0.45)
>15	23	0.28 (0.23–0.33)

GM, geometric mean.
[a] Unweighted n.
[b] Urinary cadmium concentrations (ng/ml) were divided by urinary creatinine concentrations (mg/dl) and multiplied by 100 to obtain standardized cadmium concentrations (μg/g).
[c] Weighted percent.
[d] Adjusted for age at screening (continuous), education (≤high school education, some college or associate degree, college graduate or above), and smoking status (never, former, current smoker of <20 cigarettes/day, current smoker of ≥20 cigarettes/day).
